# RICH HISTORY OF GERONTOLOGY AND GSA

**DOI:** 10.1093/geroni/igae098.1650

**Published:** 2024-12-31

**Authors:** Steven Zarit

**Affiliations:** The Pennsylvania State University, University Park, Pennsylvania, United States

## Abstract

In this presentation, Dr. Steven H. Zarit will lead a panel discussion exploring the historical underpinnings of gerontology and the Gerontological Society of America (GSA), drawing from his distinguished career and vast contributions to the field. Dr. Zarit, celebrated for his groundbreaking research on caregiver burden and stress, as well as family relationships across the lifespan, will delve into the evolution of gerontology and its profound impact on his work. Acknowledging his accolades, including prestigious awards from the GSA and the American Psychological Association, Dr. Zarit will reflect on his journey into gerontology and its intersection with seminal figures like Drs. Powell Lawton, James Birren, and Bernice Neugarten. The panel discussion will feature additional insights, drawing on personal experiences and perspectives. Through a series of questions posed by the moderator, the panelists will delve into their personal motivations, the transformative developments within gerontology, and the enduring influence of key figures in the field. Following the moderated discussion, the audience will have the opportunity to engage in an interactive question-and-answer session, fostering a dynamic exchange of ideas and insights. By drawing on the lessons of the past, this presentation aims to inspire visionary approaches to the future of gerontology informed by a panel who collectively have 145 years in the field.

